# Adjuvant interferon in the treatment of melanoma – reply

**DOI:** 10.1054/bjoc.1999.1244

**Published:** 2000-04-27

**Authors:** M R Middleton

**Affiliations:** CRC Department of Medical Oncology, Christie Hospital NHS Trust, Wilmslow Road, Withington, Manchester, M20 4BX, UK


*British Journal of Cancer* (2000) **82**(10), 1755–1758
© 2000 Cancer Research Campaign
DOI: 10.1054/ bjoc.1999.1225, available online at http://www.idealibrary.com on IDEAL®

Letters to the Editor

### Adjuvant interferon in the treatment of melanoma

**Sir**

We write in response to the editorial by MR Middleton regarding the first analysis of the intergroup E1690 trial of high- and low-dose interferon for high-risk melanoma conducted in the US between 1990 and 1995. This large trial accrued 642 patients with resectable deep primaries or node-positive disease to a three-arm trial of observation, high-dose interferon α-2b (IFN-α-2b) for 1 year or low-dose IFN-α-2b for 2 years. The editorial was submitted in February, after presentation of this trial's preliminary analysis to the ESMO in late 1998. But this study has now been presented in much greater detail to the American Society of Clinical Oncology, and is in review for publication. Middleton's editorial concluded that the high-dose regimen can not be recommended as the standard of therapy for high-risk melanoma patients; however, this conclusion is based on very incomplete information, since the data from E1690 have yet to be fully presented in a formal publication. We would like to correct a number of errors in the editorial, and urge the community to carefully consider the data of the formal E1690 trial when they are published this year.

1. In reference to differences in overall survival between the study population in the E1690 trial and the study population in the preceding E1684 trial, Dr Middleton writes, 'This unexpected difference in the results of the two studies can be explained by an improvement in the outcome of these patients kept under observation … [and] may be due to changes in the staging and surgical techniques: for example, sentinel – node mapping…'.

**Response**: The E1690 trial results differ most significantly from E1684 in regard to post-relapse survival of patients in the observation arm, and this has nothing to do with differences in staging or surgical technique. In point of fact, very few patients on E1690 were staged using sentinel lymph node biopsy, and none were assessed as node-positive based on immunochemical or reverse-transcriptase polymerase chain reaction analyses.

2. Dr Middleton also writes, 'In the earlier trial, significant differences in overall survival were only seen amongst patients with clinically apparent lymphadenopathy prior to resection.'

**Response**: Significant differences in overall survival in E1684 were seen in the intent-to-treat analysis of the *entire* population. These differences, which were statistically significant for the entire trial, were yet more significant in the histologically node-positive population, which comprised 89% of the accrual to E1684. In point of fact, the hazard ratio associated with high-dose IFN-α-2 therapy on E1684 was greatest (indicating the most improvement in survival) for patients with clinically negative but pathologically positive nodes.

We would agree that the lack of efficacy of low-dose IFN-α observed in the E1690 trial, coupled with the negative results of the WHO 16 trial of low-dose adjuvant IFN lead to a compelling conclusion that low-dose IFN-α-2b is less effective than the high-dose regimen. We would also agree that 'there seems little doubt that high-dose interferon has an impact on melanoma, and can delay the time to relapse in high risk melanoma patients.'

We believe that the consistent improvement in the continuous relapse-free survival of high-risk melanoma patients receiving high-dose IFN-α-2b seen in both E1690 and E1684 corroborates the biologic activity of this regimen. With regard to the survival benefit observed in E1684, this trial was conducted at a time when IFN-α was not available for cross-over therapy, and all patients treated on E1684 had undergone full lymphadenectomy and so had no opportunity for cross-over. This situation is distinctly at variance with E1690. In E1690, there was more than a twofold larger number of patients with clinically negative but unresected lymphatics, and these patients had the opportunity to cross-over to IFN-α therapy if they had a regional nodal recurrence. In fact, they did so in substantial numbers, asymmetrically pursuing IFN salvage therapy from the observation arm after failure in regional lymphatics. It is well recognized that regional lymph node relapse is the most frequent site of relapse in melanoma patients who have not undergone lymphadenectomy, and this occurrence in more than 40% of the total number of patients treated on E1690, provided an opportunity for a confounding second exposure to IFN at relapse. Such was not the case in E1684. A retrospective analysis of salvage therapy demonstrated significantly greater numbers of patients from the observation arm than from the high-dose IFN arm were treated with IFN-α salvage therapy. This provides a plausible explanation for the differences between the E1684 and E1690 trials in terms of post-relapse and overall survival outcome.

We would urge the readership to review the data from E1690 when published in *J Clin Oncology* and to draw their own conclusions. It is our responsibility as oncologists to present the data regarding high-dose IFN-α-2b to our high-risk melanoma patients fully and in a balanced fashion. Ultimately, it should be the patient's choice to accept or reject treatment.

*JM Kirkwood,*
*Department of Medicine,*
*University of Pittsburgh Medical Center and*
*University of Pittsburgh Cancer Institute,*
*Pittsburgh, PA 15213–2582, USA*

DOI: 10.1054/ bjoc.1999.1244, available online at http://www.idealibrary.com on IDEAL®

### Adjuvant interferon in the treatment of melanoma – reply

**Sir**

Although accepted for publication in February 1999 the editorial in question was amended in proof to take into account Dr Kirkwood's presentation to the ASCO congress, as is made clear in the text. Dr Kirkwood suggests that there are a number of 'errors' in the editorial, but provides no evidence for these, although there is a difference in our interpretations of the *available* data, which is incomplete. In the absence of the definitive report on E1690 it is reasonable to *speculate* that stage migration and changes in surgical technique might explain the difference between the results and those of E1684. Dr Kirkwood points out one such change in that twice as many patients had clinically negative unresected lymphatics in the later study. Only he can appreciate the role, if any, of sentinel node biopsy until the E1690 results are published.

The editorial acknowledges that overall survival was improved by high-dose interferon (HDI) in the original study. Amongst the four sub-groups analysed in the trial report only patients with clinically apparent lymphadenopathy showed a statistically significant improvement in survival. It is fair to say that the group with clinically negative histologically positive nodes was too small to allow interpretation of interferon's efficacy. However, to maintain that this is the population with the most to gain from HDI on the basis of a 34 patient sample is tenuous, and not a claim Dr Kirkwood made in the original report on E1684.

We are agreed that HDI is active in melanoma, and that cross-over salvage therapy is the most plausible explanation for the conflicting results obtained. Given its toxicity and the suggestion that it remains effective at second relapse further work is required to pinpoint the role of HDI in melanoma. Thus, it is not possible to commend HDI as the standard adjuvant therapy in melanoma at high risk of recurrence. Indeed, various cooperative groups are pursuing trials in this field in which the control arm is observation only and Schering Plough recently abandoned their study in resected stage III melanoma in which HDI was the control arm.

Patients should undoubtedly be informed of the results of both trials, but only in the USA will they be permitted to accept or reject treatment. The conflicting results of the E1684 and E1690 studies mean that few purchasers in the UK currently funds HDI for melanoma at high risk of recurrence. Only patients with the means to fund treatment themselves will be able to come to their own decision. The way forward is to design and execute studies that address the issues thrown up by the imminent publication of the full E1690 results.

*MR Middleton*
*CRC Department of Medical Oncology,*
*Christie Hospital NHS Trust, Wilmslow Road,*
*Withington, Manchester M20 4BX, UK*

DOI: 10.1054/ bjoc.1999.1219, available online at http://www.idealibrary.com on IDEAL®

### Serum tissue polypeptide-specific antigen (TPS): what is its diagnostic value?

**Sir**,

We have read with interest an article of Rebhandl et al (1998) on the diagnostic usefulness of the tissue polypeptide-specific antigen (TPS) in neuroblastoma and Wilms tumour. We would like to share our clinical experience with TPS, which is less convincing than that presented, and have a comment to add on the theoretical and technical part of the paper.

Traditionally, TPA – summing fragments of (cyto)keratins 8, 18, 19 – and TPS – the soluble fragment of (cyto)keratin 18 – have been interpreted by some researchers as markers for cell proliferation (Einarsson and Rylander, 1997; Mishaeli et al, 1998). With the advent of knowledge on apoptosis it has been found that one of the central effector molecules, caspase-3, utilizes (cyto)keratin 18 but not (cyto)keratin 8 as a substrate (Caulin et al, 1997). This recent observation implies that (cyto)keratin 18 may be specifically degraded upon receiving an apoptotic stimulus, thus putatively producing a TPS-like material. We currently explore this concept on the MCF-7 breast cancer-derived cell line, which is deficient in caspase-3 (Janicke et al, 1998). Altogether, tumour markers based on detection of (cyto)keratin fragments, TPA, TPS and CYFRA21-1 may, at least to some extent, reflect degradative rather than proliferative cellular events. Apoptosis-inducing antitumour therapy (cytotoxic drugs, radiotherapy) leads to downstream activation of caspase-3 in most systems studied (Hannun, 1997). The question of cleavage products of this reaction with (cyto)keratin 18 as a substrate has not yet been addressed.

In the presence of sepsis and/or renal insufficiency, TPS values are indeed elevated. However, minor or localized infection and liver and/or multiorgan failure can also lead to elevation of TPS with either no apparent underlying malignancy or no change in stable disease, as we have repeatedly observed in our patients. The authors state, that 'these samples were, therefore, excluded' without giving specific criteria. It should be noted that TPA/TPS are fairly unspecific biomarkers and for diagnosis are of similar value as erythocyte sedimentation rate. We assume that diagnoses in these patients were based on standard techniques. In this sense, interpreting TPS as a diagnostic marker and assessing its specificity using ROC after careful *a priori* elimination of confounders seems inappropriate, since the TPS value apparently adds nothing to the diagnostic procedure. On the other hand, data from Table 1 of the paper indicate that TPS could be interpreted as a therapy response marker (Pronk et al, 1997) as long as variables (intercurrent infections, etc.) are under control. It would also be informative to include comparison with established biomarkers for neuroblastoma and Wilms' tumour (catecholamines, NSE). That 'the potential of TPA in Wilms' tumour (Ishiwata et al, 1991) have gone unnoticed in the literature', as the authors state, may merely reflect the fact that the TPA value has never contributed new and clinically relevant information.

At our institute, we performed measurement of TPA for about 8 years (approx. 5500 measurements year$^{-1}$); 7 months ago we replaced TPA with TPS Beki (Sweden) due to automation and

 © 2000 Cancer Research Campaign

